# The Spectrum of Congenital Heart Disease with Transposition of the Great Arteries from the Cardiac Registry of the University of Padua

**DOI:** 10.3389/fped.2016.00084

**Published:** 2016-09-22

**Authors:** Carla Frescura, Gaetano Thiene

**Affiliations:** ^1^Cardiovascular Pathology, Department of Cardiac, Thoracic and Vascular Sciences, University of Padua Medical School, Padua, Italy

**Keywords:** congenital heart disease, complete TGA, corrected TGA, discordant ventriculo-arterial connection, univentricular hearts

## Abstract

Transposition of the great arteries (TGA) is a cardiac condition in which the arterial trunks arise from the inappropriate ventricle: the aorta from the right ventricle and the pulmonary trunk from the left ventricle [discordant ventriculo-arterial (VA) connection]. In complete TGA, the discordant VA connection is associated with situs solitus or inversus and concordant atrioventricular (AV) connection. The hemodynamic consequence of these combined connections is that systemic and pulmonary circulations function in “parallel” rather than in “series”. The presence of situs solitus or inversus associated with both AV and VA discordant connections characterizes a different anatomical complex known as “corrected TGA.” In these hearts, the double discordance at AV and VA levels permits a normal sequence of the blood flow from the right atrium to the pulmonary artery and from the left atrium to the aorta. The systemic and pulmonary circulation in these hearts functions regularly in series, and the blood sequence is “physiologically corrected.” Thus, the term transposition, either complete or corrected, identifies two precise, different anatomical complexes, both characterized by VA discordance. However, among congenital heart disease (CHD), there are other anatomical complexes with discordant VA connection in the setting of isomeric atrial situs (right or left) or of univentricular AV connections (double inlet or absent connections). In these latter conditions, the term “transposition” is still necessary to stress that the great arteries are “transposed” in relation to the ventricular septum (aorta from the right ventricle and pulmonary trunk from the left ventricle) but certainly does not figure out the anatomical complexes named complete or corrected transposition. We reviewed the hearts with discordant VA connection of our Anatomical Collection, consisting of 1,640 specimens with CHD, with the aim to discuss the anatomy and the frequency of the anatomical variants of TGA and to clarify terminology and classification. The knowledge of the precise anatomy of these malformation are really important for clinical diagnosis and surgical planning.

## Introduction

Transposition is the condition in which the great arteries take origin from the ventricles in reverse position across (“trans”) the ventricular septum: the aorta from the right ventricle and the pulmonary trunk from the left ventricle (1). In terms of segmental sequence, this corresponds to discordant ventriculo-arterial (VA) connection (2–5).

Complete transposition of the great arteries (TGA) identifies an anatomical complex in the setting of a defined situs (solitus or inversus), in which the right atrium is connected with the right ventricle from which the aorta takes origin and the left atrium is connected with the left ventricle from which the pulmonary artery takes origin [concordant atrioventricular (AV) and discordant VA connections] (2–10). Different anatomical complexes of complete TGA can be identified according to the presence of intact ventricular septum, ventricular septal defect (VSD), or obstructions to the right or left ventricular outflow.

However, among congenital heart disease (CHD), there are other malformations in which discordant VA connections are present. In situs solitus or inversus, discordant VA connection can be found in association with discordant AV connection (so-called “congenitally corrected transposition”) (11–13) or in presence of univentricular connection (absent or double inlet connection) (14). Moreover, discordant VA connections are also present in hearts with right or left isomerism (15) with biventricular or univentricular AV connections (14).

Our aim was to identify, by reviewing the hearts of the Anatomical Collection of CHD of the University of Padua, all the cases with discordant VA connection for a description of the anatomical variants and incidence of TGA and to clarify terminology and classification.

## Material and Methods

We have reviewed the specimens of our Anatomical Collection of CHD consisting in 1,640 hearts with the purpose to identify cases with complete TGA and all others cases with discordant VA connection. Our Collection started in 1968 and consists in 1,640 heart and lungs blocks derived from routinary autopsy of patients with CHD, died naturally or after cardiac surgery. Each specimen is formalin fixed and conserved in a single jar. About 40 new specimens are added to the Collection each year. The original autopsy record has been reviewed as well. The hearts are classified according to the segmental sequential chamber analysis, and the data are collected in an electronic data base.

**Figure 1:**
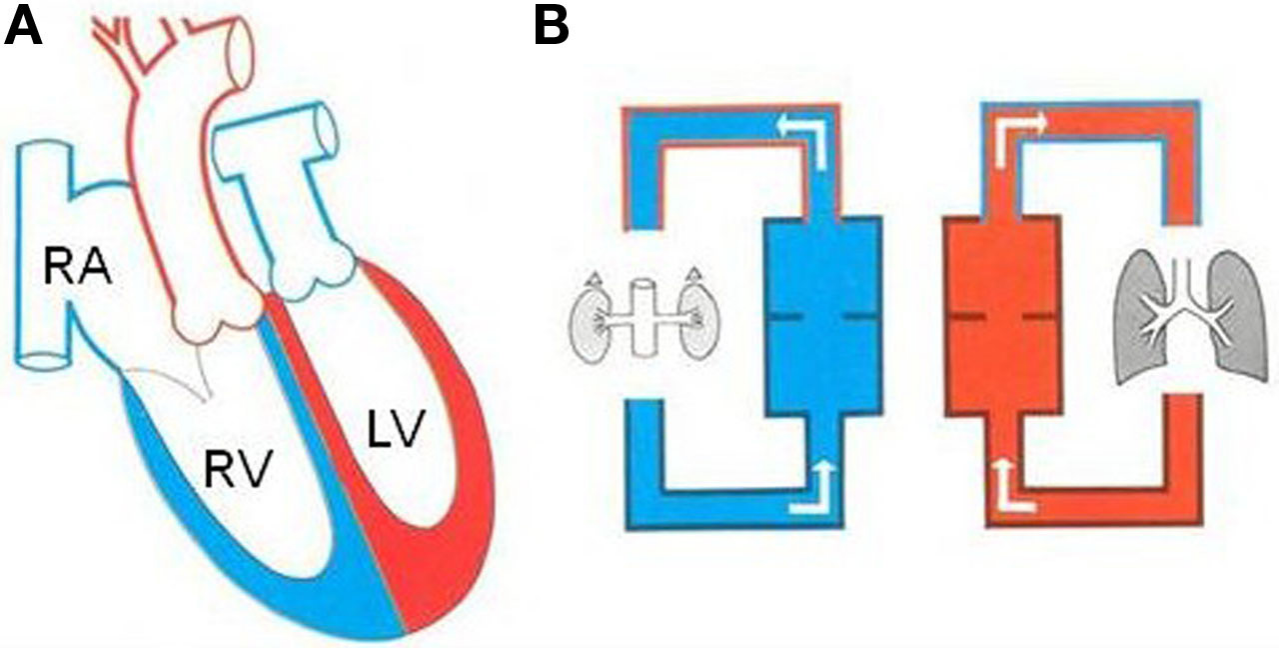
**Complete TGA in atrial situs solitus**. **(A)** Diagram illustrating complete TGA with situs solitus and concordant AV connection. **(B)** The systemic and pulmonary circulations are in parallel. LV, left ventricle; RA, right atrium; RV, right ventricle.

According to the sequential chamber classification (2–5), we classified the hearts with discordant VA connection in four groups characterized by:

- situs solitus or inversus, AV concordance: complete TGA (Figure 1),
- situs solitus or inversus, AV discordance: corrected TGA,
- situs solitus or inversus with univentricular AV connections,
- right or left isomerism with biventricular or univentricular AV connections.

The hearts with complete TGA were subdivided in four groups:

- TGA with intact ventricular septum,
- TGA with VSD,
- TGA with VSD and aortic arch obstruction,
- TGA with VSD and pulmonary stenosis.

In the last group, only hearts with severe congenital valvular or subvalvular pulmonary stenosis requiring Rastelli operation were considered.

The aortic sinuses from which the coronary arteries take origin were identified as “right hand” (sinus #1) or “left hand” (sinus #2) sinus, corresponding to the hands of an observer located in the non-facing sinus looking toward the pulmonary trunk.

## Results

177 hearts with discordant VA connection were present in our Collection of CHD (11%). The atrial situs was: solitus in 171 cases, inversus in 3, right isomerism in 2, and left isomerism in 1 (Table 1).

**Table 1:** **Discordant VA connection: atrial situs and AV connections**.

Atrial situs	AV connection (no. of cases)							
Solitus (171)	Biventricular	135	Concordant (complete TGA)	127
			Discordant (corrected TGA)	8
	Univentricular	36	Double inlet left ventricle	23
			Absent right	6
			Absent left	7
Inversus (3)	Biventricular	3	Concordant (complete TGA)	1
			Discordant (corrected TGA)	2
Right isomerism (2)	Biventricular	2		2
Left isomerism (1)	Univentricular	1	Absent left	1
Total				177

*AV, atrioventricular; TGA, transposition of the great arteries; VA, ventriculo-arterial*.

Among the hearts with situs solitus, the AV connections were concordant in 127 (complete TGA) and discordant in 8 (corrected TGA). Univentricular AV connections were present in 36 hearts due to absent connection in 13 (absent right in 6 and absent left in 7) and to double inlet left ventricle (DILV) in 23 (Table 1). No case of double inlet right ventricle and discordant VA connection was found in our series.

Situs inversus was observed in 3 hearts, 1 with concordant AV and discordant VA connections (complete TGA in situs inversus) and 2 with discordant AV and VA connections (corrected TGA in situs inversus) (Table 1).

In two cases, a right isomerism was associated to asplenia syndrome (Table 1). In both cases, the AV connections were biventricular with common AV valve.

The single case with left isomerism and polysplenia syndrome was associated with absent left AV connection (Table 1).

### Complete TGA (Concordant AV and Discordant VA Connections)

Complete TGA was found in 128 cases, 127 with situs solitus and 1 with situs inversus.

### Complete TGA in Situs Solitus

**Figure 2:**
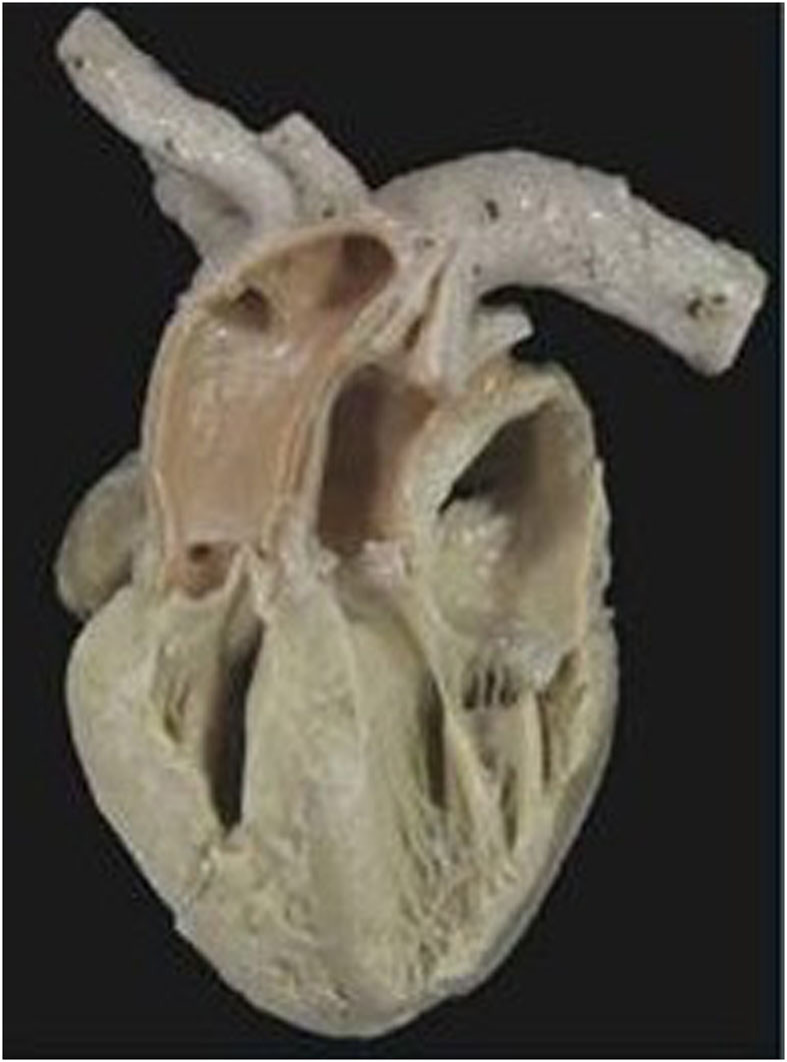
**Complete TGA with intact septum**. Anatomical specimen showing the origin of the pulmonary trunk from the left ventricle and of the aorta from the right ventricle (discordant VA connection). The ventricular septum is intact. Note the AV concordance and parallel fashion of the great arteries.

In situs solitus, complete TGA showed an intact ventricular septum in 61 cases (48%) (Figure 2) and was associated with VSD in 43 (34%). VSD and obstruction of the right ventricular outflow with aortic arch anomalies were found in 18 cases (14%) and with VSD and severe stenosis of the left ventricular outflow in 5 (4%) (Table 2).

**Table 2:** **Complete TGA**.

Atrial situs	Anatomical complex	No. of cases	Males/females	Age
Solitus (127)	TGA with IVS	61 (48%)	43/18	1 day–26 years (median 5 days)
	TGA with VSD	43 (34%)	26/17	1 day–29 years (median 3 months)
	TGA with VSD and aortic arch obstruction	18 (14%)	11/8	1 day–5 months (median 19 days)
	TGA with VSD and pulmonary stenosis	5 (4%)	2/3	4 days–3 years (median 2 months)
Inversus (1)	TGA with IVS	1	1/0	1 day
Total		128	82/45	–

*IVS, intact ventricular septum; TGA, transposition of the great arteries; VSD, ventricular septal defect*.

Eighty-two patients were males and the age at death varied from 1 day to 29 years (Table 2).

A d-ventricular loop (synonymous with right-handed topology) was present in all hearts, with levocardia in 123 and dextrocardia in 4.

**Figure 3:**
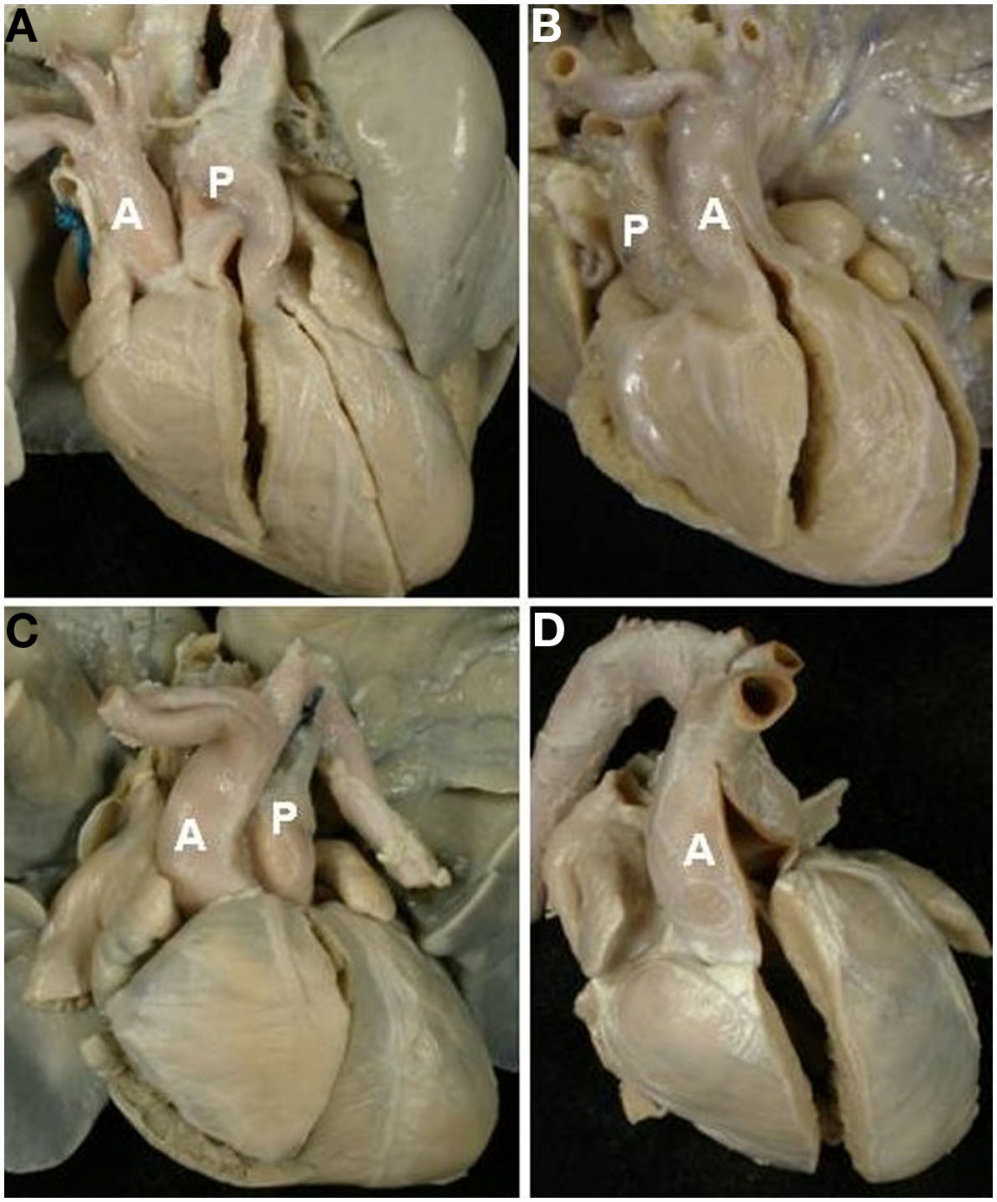
**Complete TGA: relation of the great arteries**. Aorta in right posterior **(A)**, left anterior **(B)**, right anterior **(C)**, and anterior position **(D)**. A, aorta; P, pulmonary artery.

The relations of the great arteries are reported in Table 3. The aorta was in right anterior position in 71% of cases (Figure 3C), on the right in 13%, right posterior in 2% (Figure 3A), anterior in 10% (Figure 3D), and on the left in 3 cases (Figure 3B). In all groups, there was a prevalence of right anterior position of the aorta. In hearts with aortic arch obstruction, right posterior and left anterior positions were more common (Table 3).

**Table 3:** **Complete TGA: relation of the great arteries**.

Position of the Aorta	No. of cases	TGA + IVS	TGA + VSD	TGA + VSD + Ao obstruction	TGA + VSD + Po stenosis
Right posterior	3 (2%)	–	1	2 (11%)	–
Right	17 (13%)	7 (12%)	7 (16%)	3 (17%)	–
Right anterior	91 (71%)	45 (73%)	32 (74%)	10 (55%)	4 (80%)
Anterior	13 (10%)	9 (15%)	–	3 (17%)	1
Left anterior	2	–	2	–	–
Left	1	–	1	–	–
Total	127	61	43	18	5

*Ao, aortic; IVS, intact ventricular septum; Po, pulmonary; TGA, transposition of the great arteries; VSD, ventricular septal defect*.

In all cases, the great arteries appeared parallel to one another and not spiral as in normal hearts.

**Figure 4:**
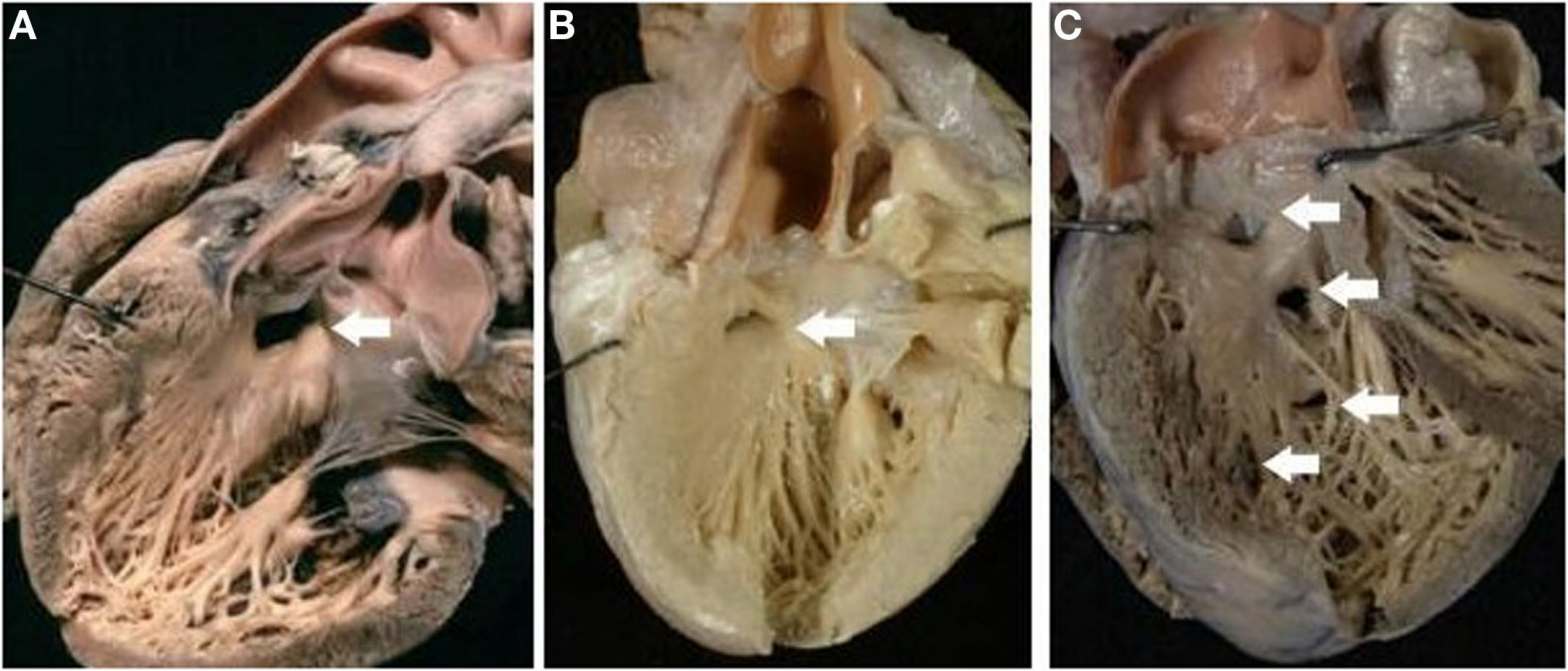
**Complete TGA with VSD**. **(A)** Perimembranous defect: the posteroinferior rim of the defect is fibrous. **(B)** Muscular defect: all the borders of the defect are muscular. **(C)** Multiple defects: a perimembranous defect and three muscular trabecular defects are present.

The VSD was present in 66 hearts and was perimembranous in 32 (48%) (Figure 4A) and muscular in 27 (41%) (Figure 4B; Table 4). In 7 cases, multiple defects were noted: in 4 cases, a perimembranous defect was associated with muscular defects (Figure 4C) and, in 3 cases, several muscular defects were present (Table 4).

**Table 4:** **Complete TGA: type of VSD**.

Type of VSD		No. of cases	TGA + VSD	TGA + VSD + Ao obstruction	TGA + VSD + Po stenosis
Perimembranous		32 (48%)	22 (51%)	6	3 (60%)
Muscular		27 (41%)	17	10 (55%)	1
Multiple	Perimembranous + muscular	4	2	2	–
	Multiple muscular	3	2	–	1
Total		66	43	18	5

*Ao, aortic; Po, pulmonary; TGA, transposition of the great arteries; VSD, ventricular septal defect*.

The muscular defect was more frequent in cases with aortic arch obstruction. Both perimembranous and muscular defect were prevalently located in the outlet portion of the ventricular septum, more rarely in the inlet or trabecular portions (Table 5).

**Table 5:** **Complete TGA: site of VSD**.

Type of VSD		No. of cases	No. of defects		
			Inlet	Trabecular	Outlet
Perimembranous		32	10	1	21
Muscular		27	5	3	19
Multiple	Perimembranous + muscular	4	1/0	1/4	2/0
	Multiple muscular	3	2	1	2
Total		66	18	10	44

*TGA, transposition of the great arteries; VSD, ventricular septal defect*.

A muscular infundibulum was subaortic in 109 cases (86%) and bilateral in 18 (14%). In no case, an isolated muscular subpulmonary infundibulum was noted. The bilateral infundibulum was more frequently present in hearts with aortic arch obstruction (Table 6).

**Table 6:** **Complete TGA: associated anomalies**.

Associated anomalies		TGA + IVS 61 hearts	TGA + VSD 43 hearts	TGA + VSD + Ao obstruction 18 hearts	TGA + VSD + Po stenosis 5 hearts
Dextrocardia		–	3	1	–
Persistent left SVC to coronary sinus		–	1	1	–
Partial anomalous pulmonary venous drainage in IVC		–	1	–	–
ASD/PFO		14/47	18/25	7/11	1/4
Appendages juxtaposition		–	7	1	1
Tricuspid valve	Hypoplastic	–	1	4	–
	Dysplastic	–	5	1	2
	Straddling	–	1	–	–
Mitral valve	Cleft	2	5	–	–
	Parachute	–	–	2	1
	Arcade	1	–	–	–
	Double orifice	–	1	–	1
	Straddling	–	2	–	–
Infundibulum	Subaortic	58	35	13	3
	Bilateral	3	8	5	2
Right outflow obstruction	Deviation of outlet septum	–	5	9	–
	Septal hypertrophy	1	–	5	–
	Hypertrophy of septoparietal bands	1	3	5	–
	Hypertrophy of TSM	–	–	3	–
	Insertion of AV valve	–	1	–	–
Left outflow obstruction	Deviation of outlet septum	–	3	–	1
	Septal hypertrophy	3	1	–	1
	Septal hypertrophy + fibrous shelf	4	3	1	1
	Fibrous diaphragm	2	2	–	1
	Anterolateral muscle band	1	1	–	–
	Insertion of AV valve	1	4	–	–
	Accessory AV tissue	–	2	–	1
Aortic valve	Bicuspid	–	–	1	–
Aortic arch	Right arch	–	2	1	–
	Coarctation	–	–	13	–
	Interruption	–	–	5	–
	Retroesophageal subclavian artery	–	1	–	–
Pulmonary valve	Dysplastic	–	7	–	3
	Unicuspid	–	–	–	2
	Bicuspid	1	5	–	2
PDA left/right		48	32/2	15	3
Bilateral PDA		–	–	1	–

*ASD, atrial septal defect; AV, atrioventricular; IVC, inferior vena cava; IVS, intact ventricular septum; PDA, patent ductus arteriosus; PFO, patent foramen ovale; SVC, superior vena cava; TGA, transposition of the great arteries; TSM, trabecula septomarginalis; VSD, ventricular septal defect*.

A malalignment of infundibular septum of different degrees was present in 37 hearts of 66 cases with VSD (56%) (rightward displacement in 29 cases and leftward displacement in 8 cases).

**Figure 5:**
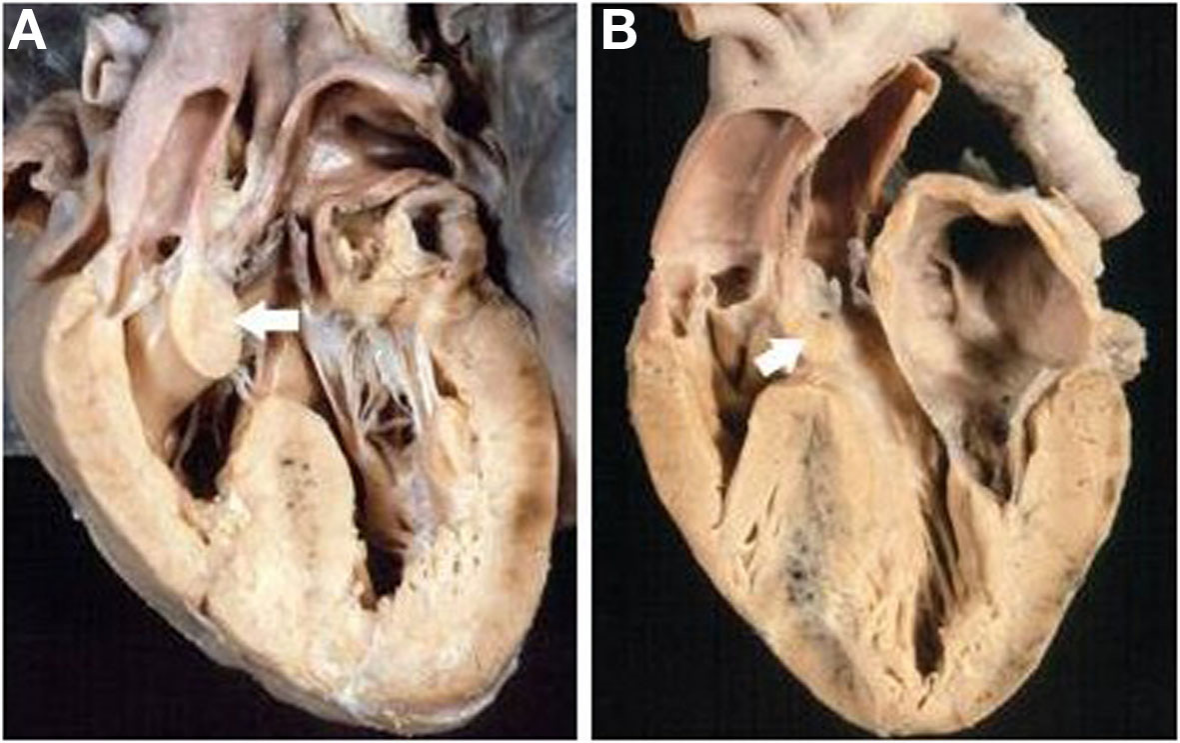
**Complete TGA with malaligment of the infundibular septum**. **(A)** The deviation to the right of the infundibular septum is the cause of obstruction of the right ventricular outlet. The pulmonary valve overrides the ventricular septum. **(B)** In this specimen, the infundibular septum is deviated to the left causing obstruction of the left ventricular outlet. An overriding aortic valve is present.

**Figure 6:**
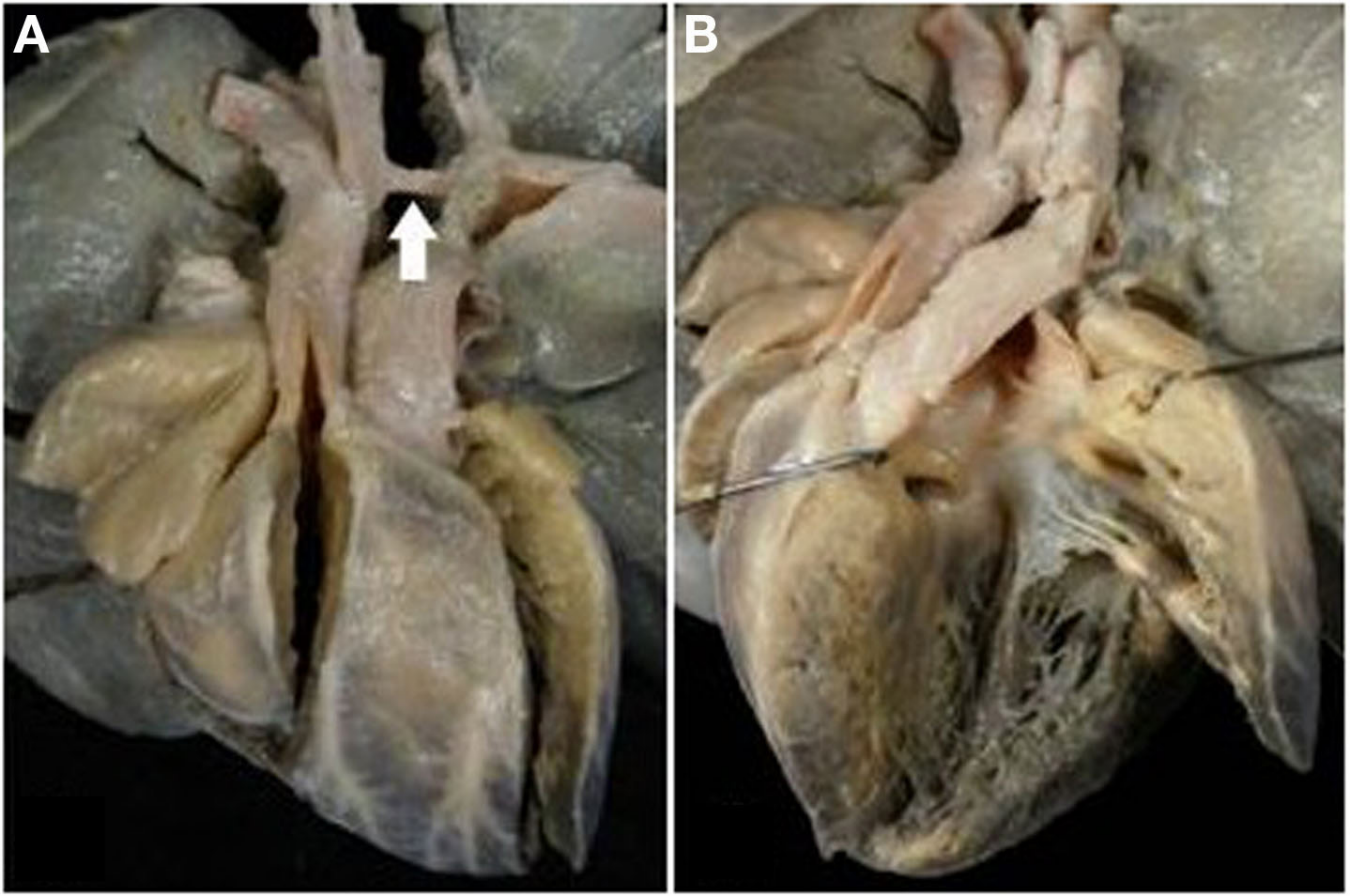
**Complete TGA with VSD and aortic arch obstruction**. **(A)** External view of the heart showing the right anterior position of the aorta and the severe hypoplasia of the aortic arch between carotid and subclavian artery (arrow). **(B)** The pulmonary artery takes origin from the left ventricle. Note the malaligment VSD with the infundibular septum deviated to the right.

Obstruction of the right ventricular outlet was the consequence of severe rightward deviation of the infundibular septum in 14 cases (Figure 5A), hypertrophy of the muscular septum in 6, hypertrophy of the septal parietal bands in 9, and hypertrophy of the trabecula septomarginalis in 3 (Table 6). Obstruction of the right outflow tract was usually associated with malformations of the aortic arch. Anomalies of the aortic arch were present in 18 cases, consisting in aortic isthmal coarctation in 13 (preductal in 6 and juxtaductal in 7) and in interruption of the aortic arch at the isthmal level in 4, and between subclavian and carotid artery in 1 (Figure 6; Table 6).

**Figure 7:**
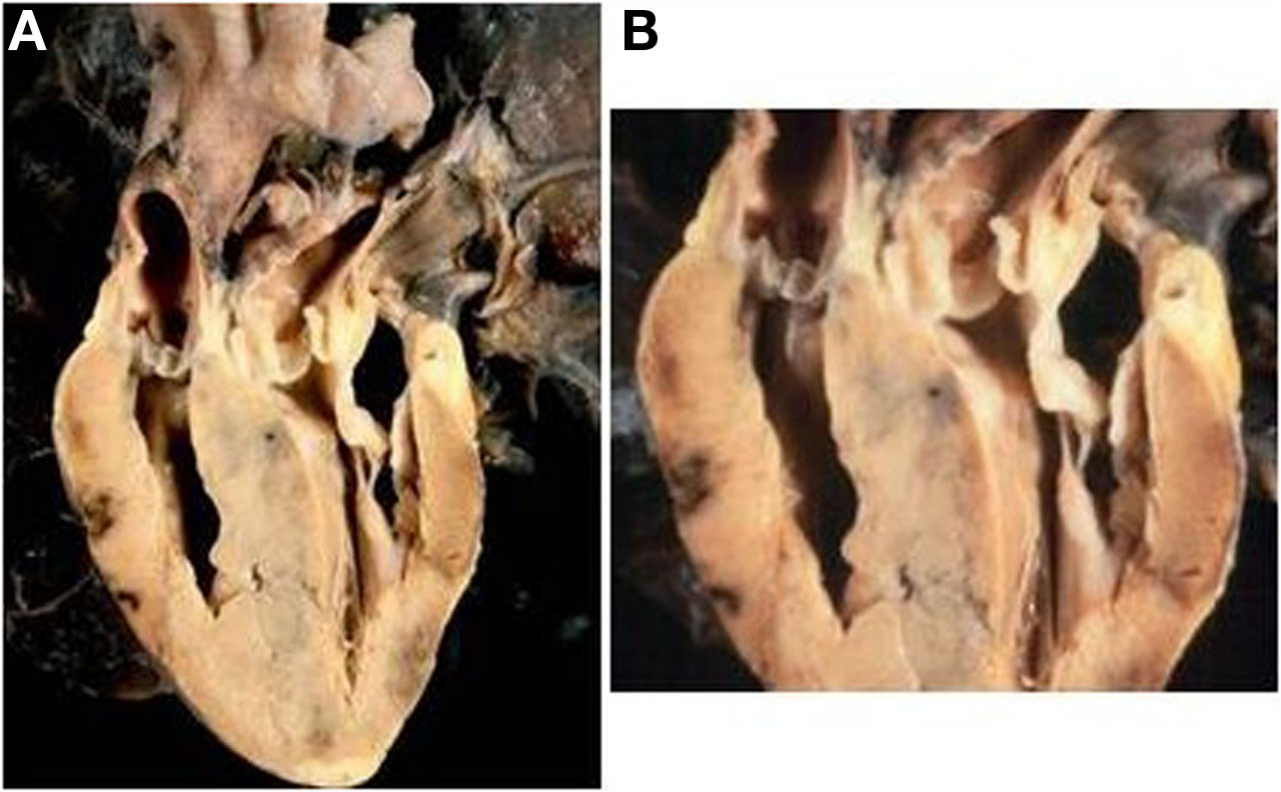
**Complete TGA with intact septum and subpulmonary stenosis**. **(A)** Echocardiographic section of the heart showing the origin of the pulmonary artery from the left ventricle and of the aorta from the right ventricle. The ventricular septum is intact. **(B)** Close up of the same specimen: note the bulging of the hypertrophic ventricular septum and the presence of a fibrous shelf due to the friction with the anterior mitral leaflet.

**Figure 8:**
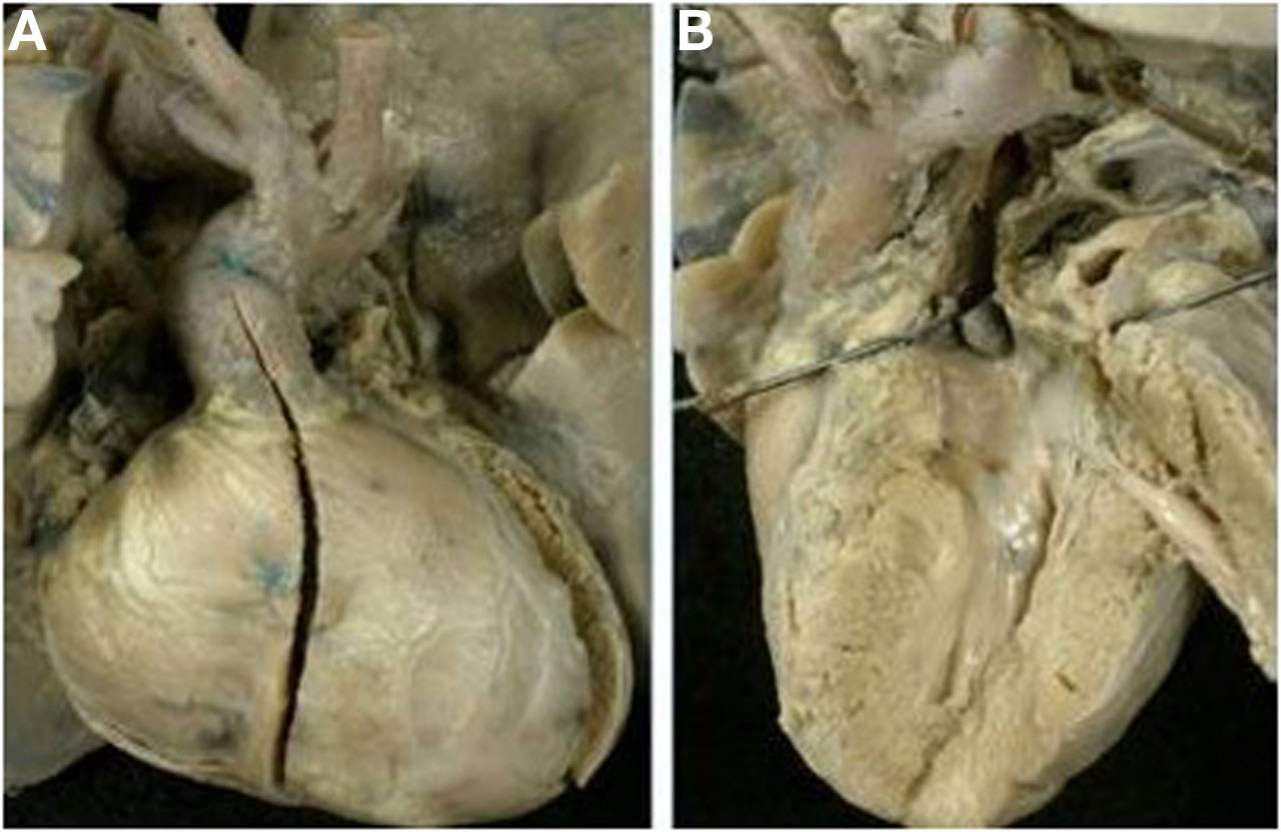
**Complete TGA with pulmonary valvular stenosis and small VSD**. **(A)** External view of the heart with right anterior position of the aorta. **(B)** View of the left outflow tract: note the subpulmonary stenosis due to the deviation to the left of the infundibular septum and the presence of unicuspid and dysplastic pulmonary valve.

Obstruction of the left ventricular outlet was due to deviation to the left of the infundibular septum in 4 (Figure 5B), hypertrophy of the interventricular septum in 14 (isolated in 5 and associated with a fibrous shelf in 9 cases) (Figures 7 and 8), and to accessory AV tissue in 2. A fibrous diaphragm was noted in 5 hearts. An anterolateral muscular band was present in 2 cases and anomalous insertion of the tensor apparatus of the mitral valve due to a cleft in the anterior leaflet in 5 cases (Table 6). Severe pulmonary valve stenosis was due to dysplastic unicuspid (2 cases) (Figure 8) or bicuspid pulmonary valve (2 cases) (Table 6).

Malformation of the mitral valve or dysplastic or hypoplastic tricuspid valves were also noted, the first associated predominantly to TGA with VSD, the latter in association with TGA with aortic obstruction. Straddling mitral valve was noted only in two hearts.

Coronary anomalies were present in 39 cases and consisted in origin of circumflex artery (CXA) from the right coronary artery (RCA) in 11, origin of left anterior descending artery (LAD) from the RCA in 8, single coronary artery in 11, and origin of both coronary arteries with separated ostia from the same sinus in 7. A pericommisural origin of coronary ostia was noted in 2 hearts (Table 7). Other associated anomalies are reported in Table 6.

**Table 7:** **Complete TGA: coronary anomalies**.

Coronary anomalies	Origin	No. of cases	TGA + IVS	TGA + VSD	TGA + VSD + Ao obstruction	TGA + VSD + Po stenosis
CXA from RCA	RCA + CXA from left hand sinus (#2), LAD from right hand sinus (#1)	11	5	6	–	–
LAD from RCA	RCA + LAD from right hand sinus (#1), CXA from left hand sinus (#2)	8	5	1	1	1
Single coronary	Single from right hand sinus (#1)	1	–	1	–	–
	Single from left hand sinus (#2)	10	4	2	2	2
Coronaries from the same sinus	Both from left hand sinus (#2)	7	2	4	1	–
Pericommissural origin	–	2	–	1	1	–
Total		39	16	15	5	3

*Ao, aorta; CXA, circumflex artery; IVS, intact ventricular septum; LAD, left anterior descending artery; Po, pulmonary; RCA, right coronary artery: TGA, transposition of the great arteries; VSD, ventricular septal defect*.

### Complete TGA in Situs Inversus

**Figure 9:**
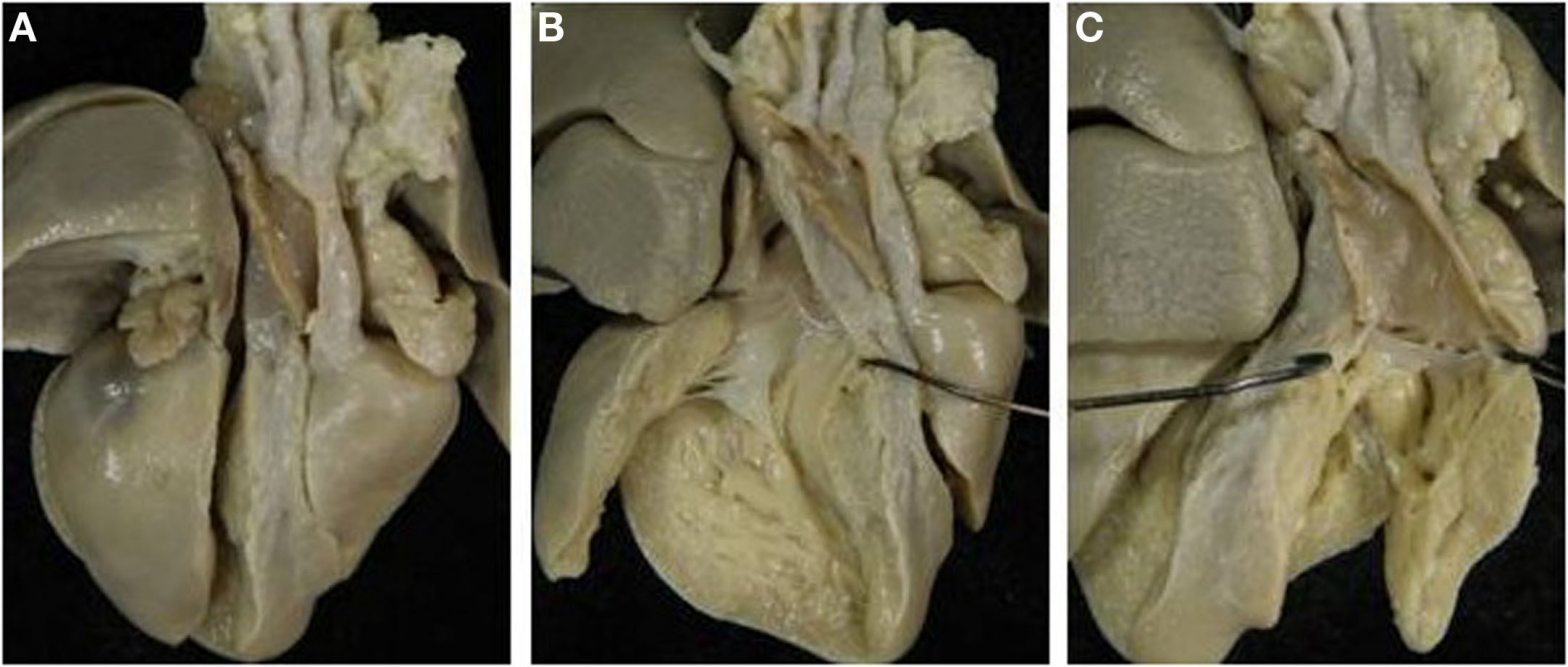
**Complete TGA in situs inversus**. **(A)** External view of the heart with dextrocardia and left position of the aorta. **(B)** The pulmonary artery takes origin from the right-sided morphological left ventricle. **(C)** The aorta takes origin from the morphological right ventricle.

Only one case of complete TGA was found associated with situs inversus (mirror image of complete TGA in situs solitus) (Table 2; Figure 9). The right sided atrium, bronchus, and lung presented a left morphology, and the left sided atrium, bronchus, and lung presented a right morphology.

Dextrocardia and l-ventricular loop (synonymous with left-handed topology) were present. The aorta was in left position, side-by-side with the pulmonary artery. An atrial septal defect (ASD), fossa ovalis type, patent right ductus arteriosus, and right aortic arch were present. The ventricular septum was intact.

### Corrected TGA (Discordant AV and VA Connections)

**Figure 10:**
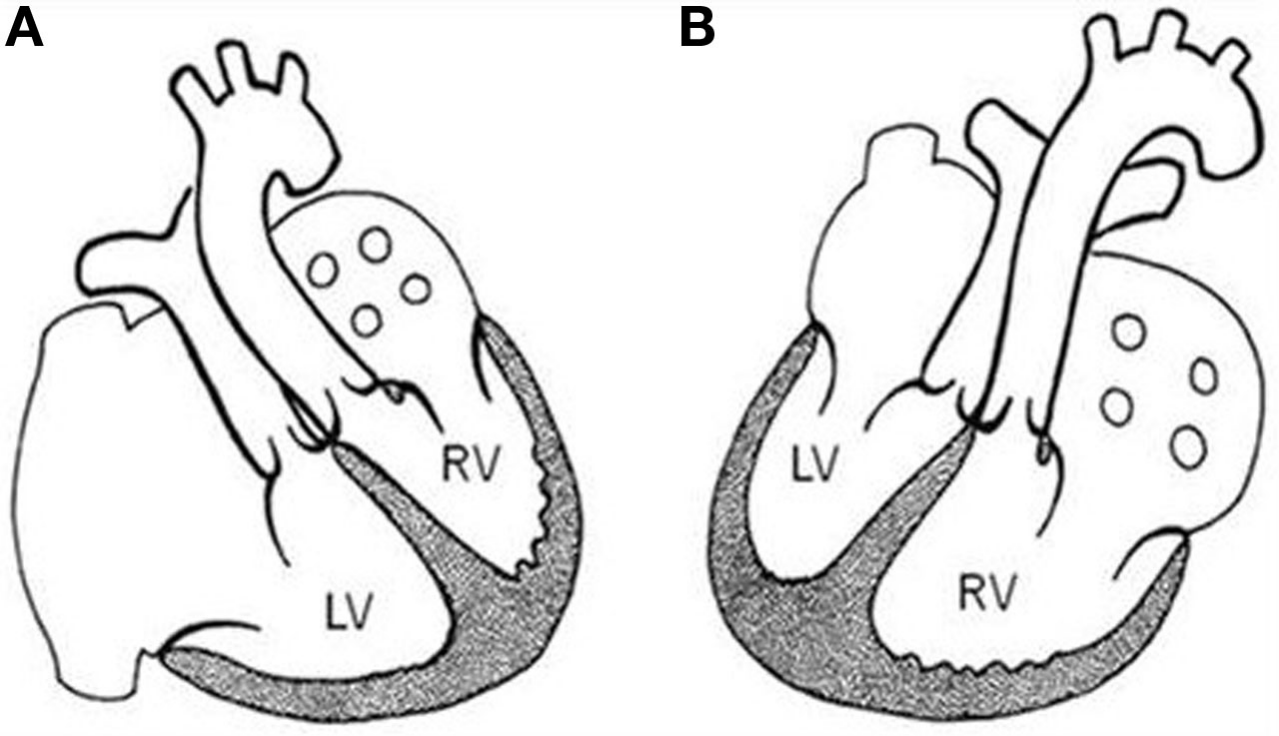
**Corrected TGA**. Schematic representation of corrected TGA (discordant AV and VA connections) in situs solitus with levocardia **(A)** and in situs inversus with dextrocardia **(B)**. LV, left ventricle; RV, right ventricle.

Discordant AV and VA connections were noted in eight cases with situs solitus (Figure 10A) and in two cases with situs inversus (Figure 10B; Table 8).

**Table 8:** **Corrected TGA**.

Atrial situs	Anatomical complex	No. of cases	Males/females	Age
Solitus (8)	TGA with IVS	5 (50%)	1/4	2 months–54 years (median 32 years)
	TGA with VSD	3 (30%)	2/1	1day–3 months (median 3 days)
Inversus (2)	TGA with IVS	1	1/0	8 years
	TGA with VSD	1	1/0	1 month
Total		10	5/5	–

*IVS, intact ventricular septum; TGA, transposition of the great arteries; VSD, ventricular septal defect*.

### Corrected TGA in Situs Solitus

Corrected TGA was present in eight patients, three males, and five females, ages varying from 1 day to 54 years (Table 8).

**Figure 11:**
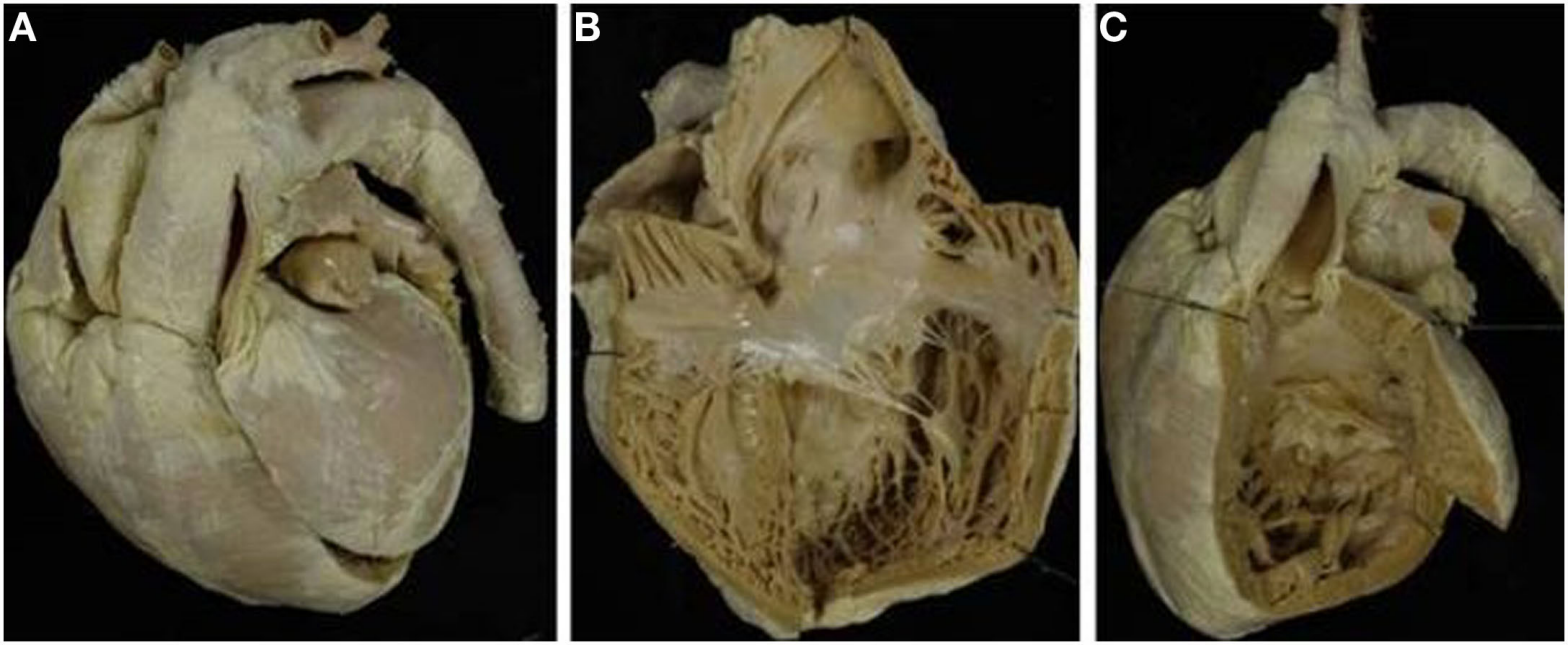
**Corrected TGA with intact septum in situs solitus**. **(A)** External view of the heart with the aorta in left anterior position. **(B)** The morphologically right atrium, characterized by the presence of the crista terminalis, connects with the morphologically left ventricle (discordant AV connection). **(C)** The aorta takes origin from the left-sided morphological right ventricle (discordant VA connection).

All hearts presented with levocardia (Table 9), l-ventricular loop, and left anterior position of the aorta (Figure 11). The pulmonary valve was imperforated in one case with ductus-dependent pulmonary circulation.

**Table 9:** **Corrected TGA: associated anomalies**.

Associated anomalies		Situs solitus (l-loop) 8 hearts		Situs inversus (d-loop) 2 hearts	
		IVS 5	VSD 3	IVS 1	VSD 1
Dextrocardia		–	–	1	–
Persistent left SVC to coronary sinus		1	–	–	1
Partial anomalous Po venous drainage in right atrium		–		–	1
Intact atrial septum		3	–	1	–
ASD/PFO		–/2	1/2	–	1/–
Tricuspid valve	Hypoplastic	–	2	–	–
	Dysplastic	1	–	1	–
	Ebstein	1	–	–	–
	Double orifice	1	–	–	–
VSD	Perimembranous	–	1	–	1
	Muscular	–	2	–	–
Sub aortic infundibulum		5	3	1	1
Bicuspid pulmonary valve		–	1	–	–
Right aortic arch		–	–	1	1
PDA (left/right)		–	3/–	–/1	1/–

*ASD, atrial septal defect; IVS, intact ventricular septum; PDA, patent ductus arteriosus; PFO, patent foramen ovale; Po, pulmonary; SVC, superior vena cava; TGA, transposition of the great arteries; VSD, ventricular septal defect*.

In 5 cases, the ventricular septum was intact whereas a VSD was noted in 3 cases (muscular in 2 and perimembranous in 1). The tricuspid valve presented a double orifice in one case, Ebstein anomaly in another and was hypoplastic in 2, and dysplastic in 1. A subaortic infundibulum was always present as well as a mitro-pulmonary fibrous continuity. Coronary arteries anomalies were noted in 3 cases: origin of LAD from RCA in 1 and single coronary artery in 2 (Table 10).

**Table 10:** **Corrected TGA: coronary anomalies**.

Coronary anomalies	Origin	No. of cases	Situs solitus	Situs inversus
LAD from RCA	RCA + LAD from right hand sinus (#1), CXA from left hand sinus (#2)	1	1	–
Single coronary	Single from left hand sinus (#2)	2	2	–
	Single from right hand sinus (#1)	1	–	1
Total		4	3	1

*LAD, left anterior descending artery; RCA, right coronary artery: TGA, transposition of the great arteries*.

Others associated anomalies are reported in Table 9.

### Corrected TGA in Situs Inversus

Corrected TGA in situs inversus was present in only 2 male patients, 1 month and 8 years old, respectively (Table 8).

Both cases presented with the morphologically right atrium and trilobed lung on the left and morphologically left atrium and bilobed lung on the right (mirror image aspect). Dextrocardia was noted in one case and d-ventricular loop in both cases. The aorta was in right posterior position in one and in right anterior position in the other. The ventricular septum was intact in one case, and a perimembranous VSD was present in the other. In the latter case, imperforated pulmonary valve with ductus-dependent pulmonary circulation was noted. Subaortic infundibulum and right aortic arch were always present. In one case, a single coronary artery was noted (Table 10). Other associated anomalies are described in Table 9.

### TGA in Univentricular Hearts (Discordant VA Connection with Univentricular AV Connection)

Discordant VA connections were present in 36 cases with univentricular AV connections with situs solitus and in 3 cases with isomerism (Table 1).

### Discordant VA Connection in Situs Solitus and Univentricular AV Connections

Among the 36 hearts with situs solitus, the AV connections were DILV in 23, absent left in 7 cases, and absent right in 6 (Tables 1, 11 and 12).

#### Double Inlet Left Ventricle

Among the 23 patients with DILV, 14 were males and 9 were females, age ranging from 4 days to 25 years (median 1 month).

All cases presented with levocardia. D-ventricular loop was observed in 18 cases and l-ventricular loop in 5 cases (Tables 11 and 12). A right anterior aorta was present in 12 cases (66%) among the hearts with d-ventricular loop, while a left anterior aorta was mostly noted in cases with l-loop (4 cases, 80%) (Table 11).

**Table 11:** **Discordant VA connection with univentricular AV connection in situs solitus: relation of the great arteries**.

Position of the Aorta	No. of cases	AV connection				
		Double inlet LV 23 hearts		Absent left 7 hearts		Absent right 6 hearts
		d-loop	l-loop	d-loop	l-loop	d-loop
Right	1	1	–	–	–	–
Right anterior	20 (55%)	12 (66%)	–	1	1	6
Anterior	7	5	1	–	1	–
Left anterior	8 (22%)	–	4 (80%)	–	4 (66%)	–
Total	36	18	5	1	6	6

*AV, atrioventricular; LV, left ventricle; VA, ventriculo-arterial*.

**Figure 12:**
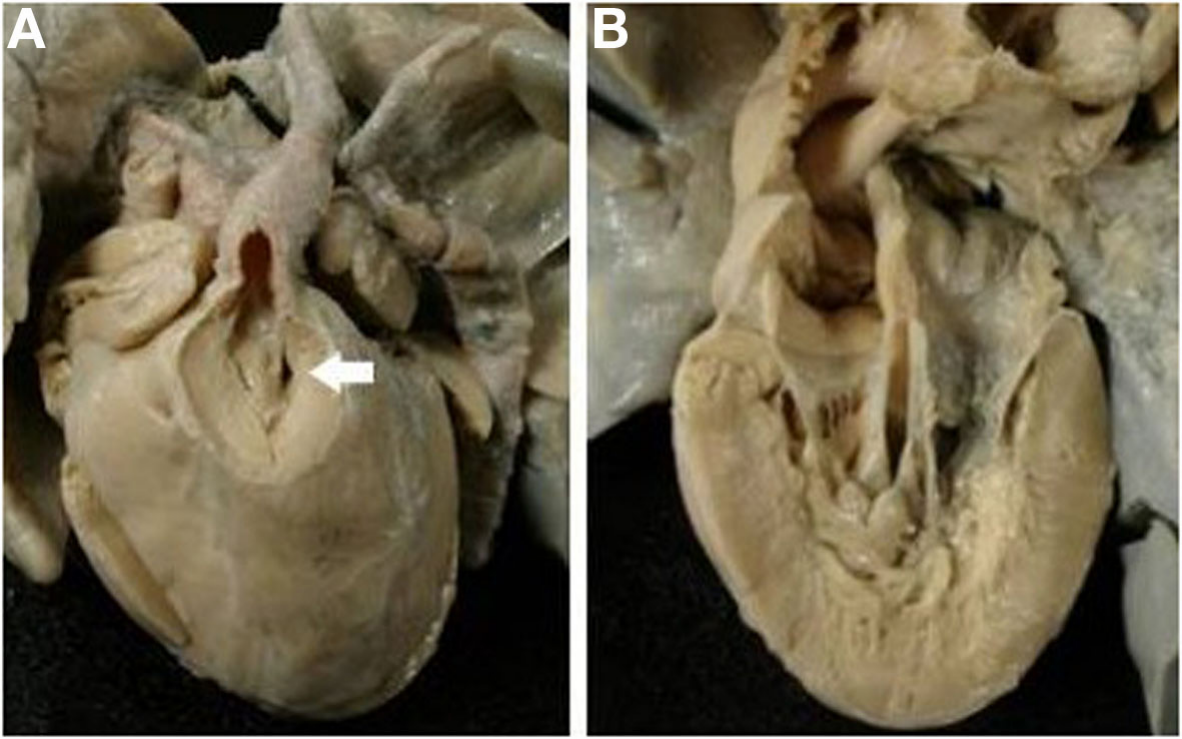
**Discordant VA connection in double inlet left ventricle**. **(A)** External view of the heart: the aorta takes origin from an anterior hypoplastic right ventricle (discordant VA connection). The arrow indicates the obstructive VSD (outlet foramen). The obstruction of the aortic arch was surgical relieved. **(B)** Two separated AV valves drain into the same ventricle of left morphology (double inlet left ventricle).

The VSD (outlet foramen) was muscular in all cases, and it was restrictive in 9 cases (Figure 12). All cases with restrictive VSD were associated with aortic arch obstruction (aortic isthmic coarctation in 6 cases and aortic arch interruption in 2 cases).

Bilateral infundibulum was present in two cases. Anomalies of the mitral valve were observed in five cases. Others associated anomalies are reported in Table 12.

**Table 12:** **Discordant VA connection with univentricular AV connection in situs solitus: associated anomalies**.

Associated anomalies		AV connection				
		Double inlet LV 23 hearts		Absent left 7 hearts		Absent right 6 hearts
		d-loop 18	l-loop 5	d-loop 1	l-loop 6	d-loop 6
Dextrocardia		–	–	–	–	2
Appendages juxtaposition		1	–	–	–	1
ASD/PFO		10/8	2/3	–/1	5/1	3/3
Mitral valve	Cleft	3	–	–	–	–
	Parachute	1	–	–	–	–
	Double orifice	1	–	–	–	–
VSD	Muscular	18	5	1	5	6
	Multiple muscular	–	–	–	1	–
Infundibulum	Subaortic	17	4	1	5	6
	Bilateral	1	1	–	1	–
Left outlet obstruction	Fibrous diaphragm	2	2	–	–	1
	Accessory AV tissue	–	–	–	2	1
Pulmonary valve	Bicuspid	1	1	–	2	–
	Dysplastic	1	–	–	–	1
Aortic valve	Bicuspid	3	–	–	–	1
	Dysplastic	1	–	–	1	–
Aortic arch	Right	–	–	–	–	–
	Coarctation	6	–	–	3	3
	Interruption	2	–	–	–	–
PDA (left/right)		9/–	3/–	1/–	4/–	6/–

*ASD, atrial septal defect; AV, atrioventricular; LV, left ventricle; PDA, patent ductus arteriosus; PFO, patent foramen ovale; VA, ventriculo arterial; VSD, ventricular septal defect*.

#### Absent Left AV Connection

Absent left AV connection was found in 7 patients, 3 males and 4 females, ages ranging from 1 day to 10 months (median 15 days).

**Figure 13:**
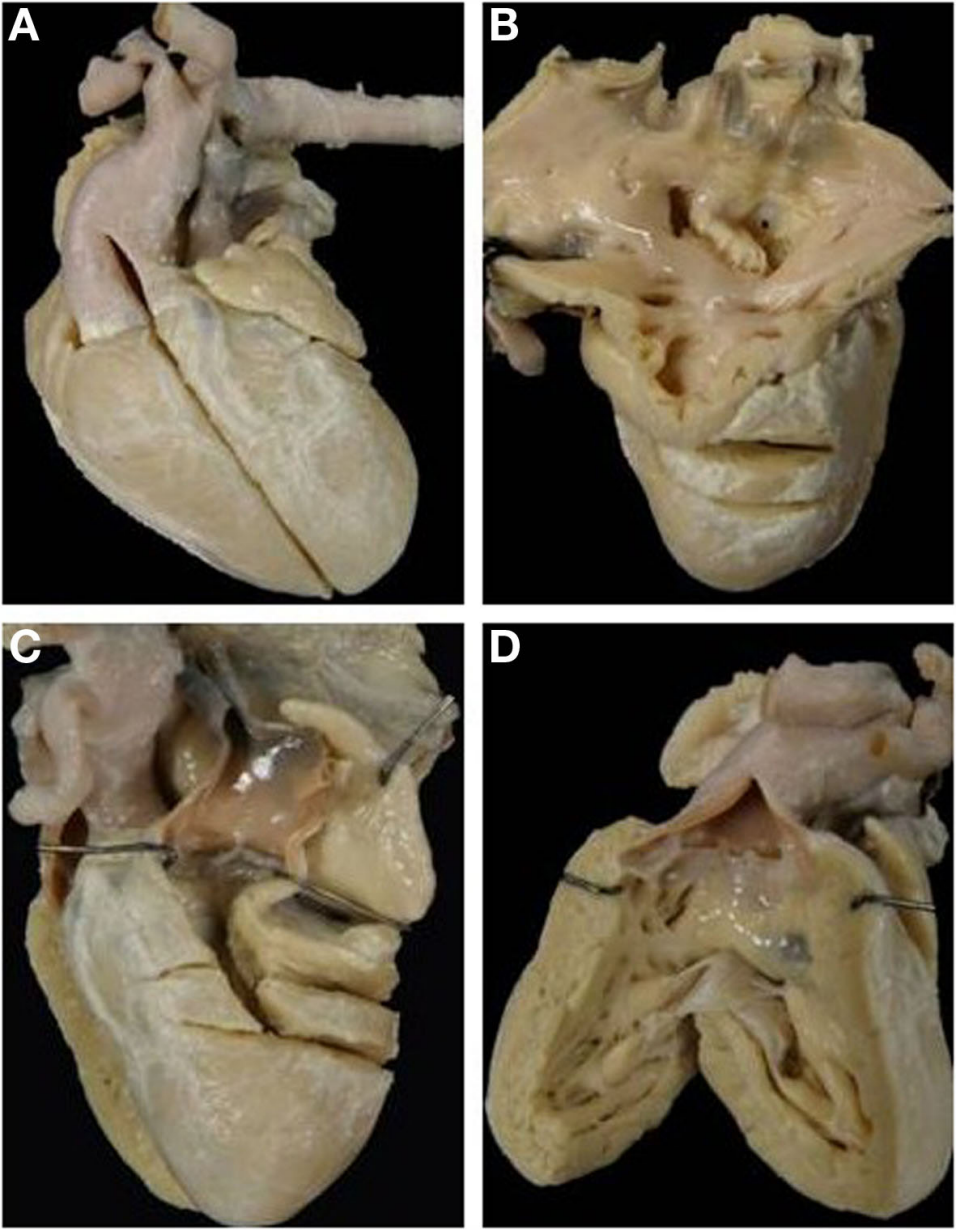
**Discordant VA connection with absent left AV connection in situs solitus (mitral atresia)**. **(A)** External view of the heart with right anterior position of the aorta. **(B)** View from the left atrium: the left AV valve is absent. **(C)** The pulmonary artery takes origin from a hypoplastic left ventricle. **(D)** The aorta originates from the right sided morphological right ventricle with a subaortic infundibulum.

Only 1 case presented a d-ventricular loop (Figure 13) while an l-loop was found in 6 cases (Tables 11 and 12). In cases with l-loop, the patent, right sided AV valve was mitral in morphology.

Levocardia was present in all cases. The aorta was in right anterior position in the case with d-loop (Figure 13) while in cases with l-loop, the aorta was prevalently left anterior (Table 11).

The VSD was muscular in all cases, and a bilateral infundibulum was found only in one case. In half of cases with l-loop, an aortic isthmic coarctation was present.

Associated anomalies are described in Table 12.

#### Absent Right AV Connection

Absent right AV valve was noted in six patients, five males and one female, ages ranging from 1 day to 6 months (median 1 day).

D-ventricular loop was present in all cases. Dextrocardia was noted in two cases and the aorta was in right anterior position in all (Tables 11 and 12).

**Figure 14:**
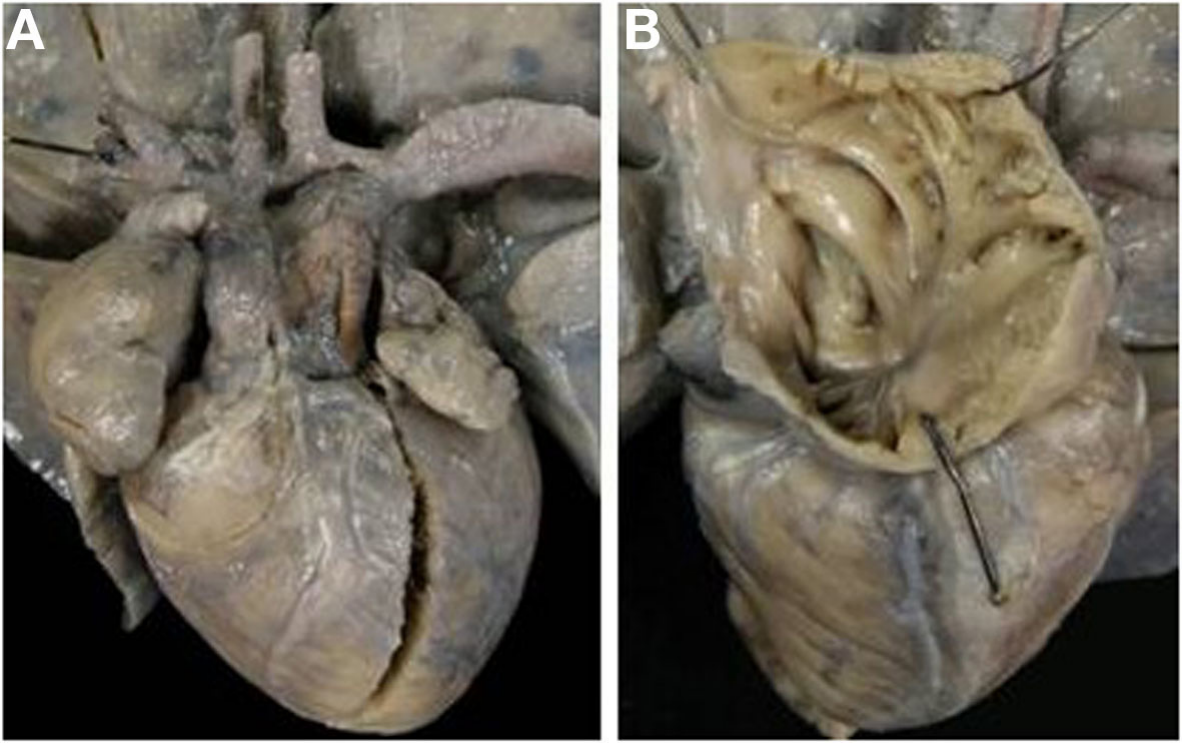
**Discordant VA connection with absent right AV connection in situs solitus (tricuspid atresia)**. **(A)** External view of the heart: the aorta is in right anterior position and severe hypoplasia of the aortic arch with aortic coarctation is present. **(B)** View of the right atrium with absence of the right AV valve.

Muscular VSD and subaortic infundibulum were present in all hearts. Aortic isthmal coarctation was noted in three cases (Figure 14).

The associated anomalies are reported in Table 12.

### Discordant VA Connection in Right and Left Isomerism

Isomerism of the atrial appendages was associated to discordant VA connection in three cases. In the two cases with right isomerism, a biventricular connection was present, while in one case with left isomerism, an absent left AV connection was found (Table 1).

All the patients were males, age varying from 1 day to 4 years. Dextrocardia, l-ventricular loop, left anterior position of the aorta, and subaortic infundibulum were noted in all cases (Table 13).

**Table 13:** **Discordant VA connection in isomerism: associated anomalies**.

Associated anomalies		Right isomerism	Left isomerism
		Biventricular AV connection (l-loop) 2 hearts	Absent left AV connection (l-loop) 1 heart
Dextrocardia		2	1
Persistent left SVC to left sided atrium		2	1
Interrupted IVC		–	1
Totally anomalous pulmonary venous drainage into right SVC		2	–
Absent coronary sinus		2	–
ASD/PFO		1/–	–/1
Common atrium		1	–
AV septal defect		2	–
Muscular VSD		–	1
Subaortic infundibulum		2	1
Left outlet obstruction	Fibrous diaphragm	1	–
Bicuspid pulmonary valve		2	1
Right aortic arch		2	1
PDA (left/right)		1/–	–/–

*ASD, atrial septal defect; AV, atrioventricular; IVC, inferior vena cava; PDA, patent ductus arteriosus; PFO, patent foramen ovale; SVC, superior vena cava; VA, ventriculo arterial; VSD, ventricular septal defect*.

In the two cases with right isomerism, an asplenia syndrome was present with bilateral trilobed lungs and symmetric short bronchi, totally anomalous pulmonary venous drainage into right SVC, absence of coronary sinus and common AV valve.

In the case with left isomerism, a polysplenia syndrome was present with bilateral bilobed lungs, symmetric long bronchi. Interruption of IVC with azygos continuation in right SVC was noted. The pulmonary veins drained into the right-sided atrium that also receives the caval returns.

Other associated anomalies are described in Table 13.

## Discussion

Since the 60s, a large pool of specimens was collected at the University of Padua concerning of children and adults with CHD, died along with the natural history or after surgery. They are kept each in a separate jar with formalin and classified according the segmental sequential chamber localization. One thousand six hundred and forty have been gathered so far. They represent an invaluable source for research and teaching and played a fundamental role for the advancement in knowledge of clinical and surgical anatomy for pediatric cardiology and surgery in our Institution as well in our Country.

The present investigation focused the issue on TGA, in order to estimate the occurrence in an autopsy series of this malformation and associated anomalies.

Herein, TGA was defined, according to sequential segmental localization of cardiac segments as discordant VA connection regardless the spatial position of the great arteries. 11% of the specimens of our Collection consists of TGA. The cases with TGA were then classified according to the atrial situs and to AV connection.

The majority of the hearts (40%) were represented by complete TGA, namely discordant VA connection in situs solitus or inversus and AV concordance, whereas only 5% consisted of so-called corrected transposition (AV and VA discordance). In a non-negligible percentage of cases (20%) TGA was observed in the setting of univentricular connection, either double inlet or absent right or left. It is noteworthy that TGA was also present in a few cases with right or left isomerism and even in situs inversus. In the latter condition, two specimens presented with corrected TGA and only one with complete TGA.

Particular attention was given here to complete TGA. The large number of cases allowed advancing a physiopathological classification: complete TGA with intact septum, with VSD, with VSD and pulmonary stenosis, and finally with VSD and aortic arch obstruction. TGA in univentricular connection was recently reported and discussed elsewhere (14).

TGA in right and left isomerism was exceedingly rare.

### Classification

In the normal heart, the aorta is in right posterior position in respect to the pulmonary trunk, and the great arteries present a spiral fashion. For long time, the presence of anterior position of the aorta was considered the landmark of TGA (16). Actually, the position of the aorta is quite variable, even if the right anterior one remains the most frequent [78% of cases of Smith et al. (17) and 71% of ours]. In rare cases, the aorta may be found in right posterior position. In these hearts, the great arteries show a normal relation, but the aorta is connected with the right ventricle and the pulmonary artery with the left ventricle (18, 19). Nowadays, the relations of the great arteries, even if important from the topographical viewpoint, are so variable that it is impossible to use as a criterion for definition (20), so that the terms l-, d-, and p-transposition are not a proper terminology.

In addition, the presence of a subaortic infundibulum was considered pathognomonic of TGA. However, in few cases, a bilateral infundibulum or exceptionally fibrous continuity between aorta and tricuspid valve have been reported.

The use of the sequential segmental classification (2–5) better allows the identification of this malformation with concordant AV and discordant VA connection in the setting of situs solitus or inversus. This anatomical complex deserves the name of “complete TGA” (6) meaning that the systemic and pulmonary blood circulation are in “parallel.”

The presence of both discordant AV and VA connections characterized a different anatomical complex known as “congenitally corrected TGA” (11–13). In these hearts, the double discordance permits a normal sequence of the blood flow from the right atrium to the pulmonary artery and from the left atrium to the aorta, even if the position of the ventricles is inverted due to a development of a l-ventricular loop in situs solitus or of a d-loop in situs inversus. The systemic and pulmonary circulations in these hearts function in series, and it is the blood sequence to be “physiologically correct.”

However, not all malformations in which the arterial trunks arise from the inappropriate ventricles are to be considered as “complete” or “corrected” TGA, because of the presence of isomeric situs or of univentricular AV connections. In these cases, on the base of the sequential classification (2–6), the description of the atrial situs and of the AV connection should be added to the term discordant VA connection.

Even if some authors (21) suggested to avoid the use of the terms “complete” or “corrected” transposition, we believe that these terms identified precisely two anatomical complexes, the first characterized by parallel systemic and pulmonary circulations and the second by a physiologically corrected circulation as a consequence of the double discordance ad AV and VA connections.

This does not influence the concept of transposition (i.e., origin of the aorta from the right ventricle and of the pulmonary trunk from the left ventricle) that remains the synonymous of discordant VA connection.

### Physiopathology

Cases with complete TGA were previously classified as “simple “or” complex” (3, 4). The first group corresponds to TGA with intact septum, characterized by the absence of other malformations (a part from a patent foramen ovale or ductus arteriosus), and the second encompasses all the forms with critical associated anomalies. We distinguish four different anatomical complexes: TGA with intact septum (equivalent to the simple form), with VSD, with VSD and aortic arch obstruction, and with VSD and pulmonary stenosis.

Transposition of the great arteries with intact septum was present in nearly half cases of our Anatomical Collection. The position of the aorta in respect to the pulmonary artery was variable and right anterior aorta was most common. No case of posterior or left aorta was noted in our series with intact septum. A bilateral infundibulum was rarely present, usually associated with side-by-side position of the arterial trunks. Hypertrophy of the ventricular septum, with or without fibrous shelf, was present in the left ventricular outflow tract causing dynamic subpulmonary stenosis.

A VSD was observed in the other half of our cases with TGA (22). Cases with obstruction of aortic arch or severe pulmonary stenosis were almost always associated with a VSD.

As in the isolated form, the VSD can be classified as perimembranous (characterized by fibrous posteroinferior rim of the defect), or muscular (exclusively muscular borders), or subarterial (the roof is formed by semilunar valves).

In our series, there was a balanced distribution between perimembranous and muscular defects even if a dominance of perimembranous location has been reported (23). Sometimes, muscular and perimembranous defects were present in the same heart as well as multiple muscular defects. The defects were prevalently located in the outlet septum. In some hearts, the VSD was the consequence of malalignment of the infundibular septum with the trabecular component of the muscular septum, and the posteroinferior border of the defect was membranous or muscular.

The anatomy of complete TGA with aortic arch obstruction was so distinctive as to be considered a separate nosographic entity among complete TGA (24–26). These hearts were anatomically different, both for position of the aorta (right posterior or left anterior), prevalence of muscular VSD, presence of bilateral infundibulum, and mild or moderate hypoplasia of the right ventricle. The aortic valve was rarely affected, and the subvalvular obstruction was mostly muscular due to rightward deviation or hypertrophy of the infundibular septum, hypertrophy of the septoparietal bands or of the trabecula septomarginalis (24–26). The malformations of the aortic arch were present in form of isthmal coarctation or interruption (at the isthmus or between carotid and subclavian artery) (25).

The rightward displacement of the infundibular septum resulted in pulmonary artery overriding. Depending on the extent of the pulmonary overriding, a spectrum does exist among hearts with discordant AV connection and hearts with double outlet right ventricle with subpulmonary defect (so-called Tausig–Bing anomaly). An accurate valuation of the degree of overriding is required in these cases, and the VA connection is discordant only when more than 50% of the pulmonary valve is aligned to the left ventricle (2–5).

The types of left outflow tract obstruction in complete TGA were the same found in hearts with concordant VA connections. The stenosis was located at subvalvular or valvular level (unicuspid, bicuspid, or dysplastic valve) (27, 28). The subvalvular stenosis was caused by hypertrophy and bulging of the muscular ventricular septum, in isolation or associated to a fibrous shelf. This type of obstruction is mostly dynamic and does not necessary require surgical relieve. Anomalous attachment or straddling of the mitral valve, accessory AV tissue, discrete fibrous ring, and anterolateral muscle bundle; all were causes of subpulmonary obstruction.

Anomalies of the mitral valve (cleft, parachute, double orifice…) were noted (29–31), complicating the surgical repair. Hypoplastic or dysplastic tricuspid valves were present mostly in cases with aortic arch obstruction (32). In rare cases, straddling or overriding AV valves are described. If the degree of AV straddling in complete TGA is less than 50%, AV connection is still considered concordant.

No case of complete TGA and AV septal defect was present in our Anatomical Collection confirming that basically complete TGA is a malformation of the arterial pole. Only in two cases with right isomerism, we found a deficient AV septation. We have never observed in situs solitus or inversus major malformation of the AV septation or of the venous pole (anomalous venous drainage). This is the reason why we consider complete TGA as an isolated malformation of the ventricular outlets.

Juxtaposition of the atrial appendages (33) can be present and influence the surgical correction.

With the advent of the arterial switch operation for the repair of complete TGA, the presence of coronary anomalies has become more relevant (34–40). In the hearts of our Collection, even if in presence of anomalous origin, all the coronary arteries arose from the sinuses adjacent to the pulmonary trunk. The presence of one coronary artery running in front of the subaortic infundibulum or in between the aortic and pulmonary trunk or burrowing through the aortic wall crossing a commissure can complicate the surgical repair.

In the absence of associated defects, patients with corrected TGA are asymptomatic and usually can reach the adult age. In these asymptomatic patients, the death can be consequence of cardiac AV block, and sudden death can be the first manifestation. In the presence of associated cardiac defects, such as VSD, tricuspid valve malformation, or pulmonary stenosis, corrected TGA may become symptomatic early in infancy.

The left position of the aorta was considered, for long time, the marker of this malformation, but even if this morphological feature is frequent, the l-position of the aorta may be present in some cases of complete TGA with situs solitus, and it is the rule in cases of complete TGA in situs inversus. This is another good reason to rule out the term l-transposition.

Double inlet left ventricle was frequently associated with discordant VA connection (23 hearts among 29 in our Collection). Restrictive VSD (outlet foramen) with aortic coarctation was frequently observed. No case of double inlet right ventricle has been observed in association with TGA.

Discordant VA connection is relatively rare in hearts with absent right or left AV connection.

In our experience, DORV or single aortic outlet (pulmonary atresia) was frequently observed in right isomerism while single pulmonary outlet (aortic atresia) may be present in left isomerism.

## Ethics Statement

The study does not refer to living patients. The collection of specimens is allowed by Italian Legislation, and the autopsy procedure is performed without the need of family consent.

## Author Contributions

CF: conceived and designed the research, drafted the manuscript, analyzed and interpreted the data. GT: conceived and designed the research, corrected the draft, handled funding and supervision.

## Conflict of Interest Statement

The authors declare that the research was conducted in the absence of any commercial or financial relationships that could be construed as a potential conflict of interest.

## Abbreviations

Ao, aortic; ASD, atrial septal defect; AV, atrioventricular; CXA, circumflex artery; CHD, congenital heart disease; DILV, double inlet left ventricle; IVC, inferior vena cava; IVS, intact ventricular septum; LAD, left anterior descending artery; LV, left ventricle; PDA, patent ductus arteriosus; PFO, patent foramen ovale; Po, pulmonary; RA, right atrium; RCA, right coronary artery; RV, right ventricle; SVC, superior vena cava; TGA, transposition of the great arteries; TSM, trabecula septomarginalis; VA, ventriculo arterial; VSD, ventricular septal defect.
